# Study protocol for a multicenter investigation of reablement in Norway

**DOI:** 10.1186/s12877-015-0108-y

**Published:** 2015-09-15

**Authors:** Eva Langland, Hanne Tuntland, Oddvar Førland, Eline Aas, Bjarte Folkestad, Frode F. Jacobsen, Ingvild Kjeken

**Affiliations:** Centre for Care Research Western Norway, Bergen University College, P.O Box 7030, 5020 Bergen, Norway; Department of Nursing, Bergen University College, P.O Box 7030, 5020 Bergen, Norway; Departement of Occupational Therapy, Physiotherapy and Radiography, Bergen University College, P.O Box 7030, 5020 Bergen, Norway; Haraldsplass Deaconess University College, Ulriksdal 10, 5009 Bergen, Norway; Institute of Health and Society, Research Centre for Habilitation and Rehabilitation Models and Services (CHARM), Faculty of Medicine, University of Oslo, P.O Box 1089, Blindern, 0318 Oslo Norway; Department of Health Management and Health Economics, University of Oslo, P.O Box 1089, Blindern, 0318 Oslo Norway; Uni Research Rokkan Centre, P.O Box 7810, 5020 Bergen, Norway; Diakonhjemmet Hospital, National Advisory Unit on Rehabilitation in Rheumatology, P.O. Box 23, Vinderen, 0319 Oslo Norway; Oslo and Akershus University College of Applied Sciences, Program of Occupational Therapy, Prosthetics and Orthotics, P.O. Box 4, St. Olavs plass, 0130 Oslo Norway

## Abstract

**Background:**

Reablement is a promising new rehabilitation model, which is being implemented in some Western countries to meet current and future needs for home-based services. There is a need for further investigation of the effects of reablement among community-dwelling adults in terms of clinical and economic outcomes. This study will investigate the effectiveness of reablement in home-dwelling adults compared with standard treatment in terms of daily activities, physical functioning, health-related quality of life, coping, mental health, use of health care services, and costs.

**Methods/Design:**

The study is a multicenter controlled trial. In total, 44 Norwegian municipalities will participate, including eight municipalities as a control group. For three municipalities with two zones, one will be assigned to the control group and the other to the intervention group. The experimental group will be offered reablement and the control group standard treatment. The sample will comprise approximately 750 participants. People will be eligible if they are home-dwelling adults, understand Norwegian, and have functional decline. Participants will be assessed at baseline, and after 10 weeks, 6 months, and 12 months. The primary outcome will be activity and participation measured by the Canadian Occupational Performance Measure. Physical functioning will be measured by the Short Physical Performance Battery and health-related quality of life by the European Quality of Life Scale. Coping will be measured by the Sense of Coherence questionnaire and mental health by the Mental Health Continuum Short Form. Costs will be generated based on registered working hours in different professions. Data analyses will be performed according to intention to treat. Univariate analysis of covariance will be used to investigate differences between the groups at baseline and the end of intervention. The data will be organized into two levels using a multilevel structure, i.e., individuals and municipalities, which will be analyzed using linear mixed-effects models. The working hours data (panel data) will be analyzed with random mixed-effects regression models. The cost-effectiveness of reablement will be evaluated according to the incremental cost-effectiveness ratio and uncertainty will be explored via the bootstrap method.

**Discussion:**

The findings will make an important contribution to knowledge of rehabilitation approaches for community-dwelling adults.

**Trial registration:**

The trial was registered at ClinicalTrials.gov on October 24, 2014, identifier: NCT02273934.

## Background

Similar to many countries around the world, the Norwegian population is ageing [1]. As more people get older, many will require assistance in order to age in place as well as to be active and participate in society. Combined with an expected shortage of health care personnel, this situation will present a challenge to the health care system in years to come [1], which has increased interest in rehabilitation services. Reablement has become an emerging model in the rehabilitation services for community-dwelling older adults experiencing functional decline [2]. The overall aim of this model is to promote everyday competence and independent functioning in daily activities among home-living people in need of rehabilitation.

Reablement is an intensive, multidisciplinary, multicomponent, person-centered, home-based type of rehabilitation, where ordinary activities of daily living are used for rehabilitative purposes [3]. The main focus is on enhancing the performance of activities that are perceived as significant and meaningful in the daily life of each individual where health care providers are organized into multidisciplinary teams, which work together with the person toward their activity goals. The intervention is intensive and occurs in the person’s home or local community.

The outcomes in terms of the effects of reablement comprise individual health outcomes, health care service utilization, and cost-effectiveness. For individual health outcomes, the effects of reablement on the performance of personal activities of daily living (PADL) have been summarized in a systematic review [4], where the authors concluded that there is limited evidence that reablement can reduce the dependency of home-care service users during PADL. However, the results of primary studies are inconsistent. In some studies, the results showed that reablement improved instrumental activities of daily living [5–7], safety [7], physical function [6–8], and health-related quality of life (HRQoL) [9, 10], whereas others found no significant improvements in safety [5], social support [8], physical function [5], or HRQoL [5, 7].

In terms of health care service utilization, the results of a recent study showed that the participants in a reablement group required fewer home care hours, were less likely to be approved for a higher level of aged care such as nursing homes, and less likely to be in need of emergency department treatment compared with participants in a conventional care group [11]. It was also shown that people who received reablement were less likely to need personal care services [5, 12] and to be readmitted into hospital [3]. Thus, these results indicate that reablement may reduce the need for home care and other health care services.

The results are conflicting regarding the cost-effectiveness of reablement. In particular, there were no significant differences between the groups in terms of cost savings in a large clinical controlled trial [10], but the results of two other studies showed that the health and home care costs of reablement were lower than the costs of conventional home care [11, 12].

In summary, there has been insufficient research into the effects of reablement, and the results of research based on individuals and cost outcomes are inconsistent. Therefore, more research is needed to increase our knowledge of the effects of reablement.

### Purpose and research questions

The main aim of this study will be to investigate the health effects and cost-effectiveness of reablement compared with standard treatment in home-dwelling adults experiencing functional decline. The following research questions are posed.

- What is the effect of reablement on performance and satisfaction with performance of daily activities in home-dwelling adults?
- What is the effect of reablement on HRQoL and physical functioning among home-dwelling adults?
- What is the effect of reablement on coping as a sense of coherence and positive mental health among home-dwelling adults?
- What is the cost-effectiveness of reablement compared with standard treatment?

## Methods/design

### Design and setting

**Fig. 1:**
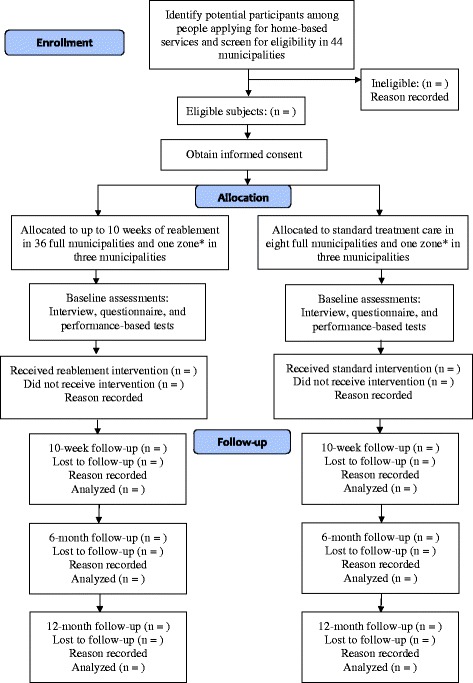
Flow diagram showing the study protocol. *Three municipalities are each divided into two zones, where one zone acts as a control group and the other as an intervention group

This will be a large multicenter clinical controlled trial. The study will be conducted in primary care settings in 44 of the 428 Norwegian municipalities, including eight municipalities that function as a control group; for three municipalities with two zones, one will be assigned to the control group and the other to the intervention group (see Fig. 1 for flow diagram). The allocation of zones to the intervention or control group was based on practical reasons and decided by the municipality. The municipalities included represent 17 % of the total population in Norway. The experimental group will be offered reablement and the control group standard treatment. The sample will comprise approximately 750 participants, who will be assessed at baseline and after 10 weeks, 6 months, and 12 months.

The protocol follows the Standard Protocol Items: Recommendations for Intervention Trials (SPIRIT) 2013 statement [13], which defines standard protocol items for clinical items, and it has been registered with ClinicalTrials.gov (October 24, 2014, identifier: NCT02273934).

### Participants and eligibility criteria

People will be eligible if they are home-dwelling, over 18 years of age, understand Norwegian, and have experienced a functional decline. Participants will be excluded if they are in need of institution-based rehabilitation or nursing home placement, terminally ill, or cognitively reduced. The inclusion criteria are not restricted to older adults, but it is expected that this age group will comprise the majority of participants.

### Interventions

#### Reablement

Reablement is an intensive, interdisciplinary intervention. In general, the intervention lasts for a period of 3–10 weeks. To enable and enhance activity performance, it is crucial to acknowledge that the person is the expert on her/his own life. The main focus is to establish a dialog to identify activities that the individual perceives as meaningful to work on or to improve. The intervention is targeted to achieving these activity goals. Therefore, the patient-specific Canadian Occupational Performance Measure (COPM) will be used as part of the baseline assessments to give directions for the modeling of the reablement intervention. The COPM interview and scoring start with the following question: “What are the most important activities in your life now?” During the COPM assessment, the participant will define up to five activity goals that are essential to her or him. Based on these goals, a rehabilitation plan will be developed to promote a match between the activities and goals identified by participants, and professional initiatives. Intensive attention will be given to encourage participation and stimulate daily training for the participants, including performing their daily tasks. Individual tailoring is a major principle of reablement, so the content of the intervention will vary among participants, although the basic features are the same. Table 1 gives an overview of the content of the intervention, including fundamental principles and key components.

**Table 1:** Content of the reablement intervention

Fundamental principles	Process, key components
• The rehabilitation period will be a maximum of 10 weeks.	• *Training in daily activities* such as personal hygiene, climbing stairs, family visits, cleaning the house, being able to write, or walking indoors/outdoors.
• A person-centered, resource-oriented, and interdisciplinary approach will be applied.	
• An occupational therapist, physiotherapist, nurse, or social educator will conduct an interview using the Canadian Occupational Performance Measure to identify activity limitations and participation restrictions. Based on the identified activity goals, a rehabilitation plan will be developed together with the participant. Next, an integrated multidisciplinary team with shared goals will collaborate with the participant throughout the whole rehabilitation period.	Optimizing performance through intervention components such as adaptations of activities and the environment.
	• *Exercise programs* such as performing exercises to improve strength, balance, or fine motor skills. The exercises will be incorporated into daily routines and the participant will be encouraged to train on their own.
• The rehabilitation will involve repetitive training and multiple home visits by health care personnel, who will be present during daily training to build confidence and relearn skills.	
• All health care personnel will stimulate the participant in self-management and self-training.	

#### The control intervention: standard treatment

The control group will receive standard treatment. In contrast to reablement, the standard treatment will not be time limited and thus it may continue after 10 weeks. Standard care often comprises compensating help and the content of the compensating help will be delivered according to the applications made by the participants. This may be personal or practical assistance, meals on wheels, safety alarm, or assistive technology. However, it may also involve rehabilitation by health professionals such as nurses, occupational therapists, and physiotherapists. This means that the standard treatment or care will vary among participants and municipalities.

### Training of the intervention providers and contact persons in each municipality

We will arrange a two-day course where representatives from all 44 municipalities will receive training in performing the data collection procedures, as well as designing and delivering the intervention. On the first day, an expert on COPM will give lectures and instructions, including practical exercises. On the second day, the principal investigator and project coworker will present a review of the data collection procedures. Each municipality will have a contact person who will be responsible for the different procedures employed by the project, including data collection. Each contact person will receive an evaluation pack, including all of the procedures and data collection instruments. They will also be encouraged to use videos to demonstrate how to perform the COPM interviews and the physical function tests, which comprise part of the baseline assessments. It will be important to ensure compliance with the intervention and the data collection routines. Thus, individual supervision will be provided by telephone during the intervention and data collection period, and the contact persons and health care providers will be encouraged to contact the principal investigator if they need to discuss different issues related to the project.

### Outcomes

Reablement is based on a holistic approach, so different outcomes will be used to detect potential changes in a variety of outcomes, including mental health and coping as a sense of coherence. The outcomes will also include cost outcomes such as consumption of various home-based services. These outcomes will be registered on a weekly basis during the first 6 months after inclusion. Table 2 provides an overview of the different outcomes that will be measured.

**Table 2:** Summary of measures to be collected

Outcome	Data collection instrument and scale	Time points
Primary outcome measures		
Activity performance	Canadian Occupational Performance Measure. Scale 1–10, 1 is low performance	t1, t2, t3, t4
Satisfaction with activity performance	Canadian Occupational Performance Measure. Scale 1–10, 1 is low satisfaction	t1, t2, t3, t4
Secondary outcome measures		
Physical Performance Test	Short Physical Performance Battery, which has three parts: 1) standing balance, 2) walking four meters at regular pace, and 3) ability to stand up and sit rapidly five times. Each part is scored from 0–4 points and the total score = 0–12 points, where 0 is low performance	t1, t2, t3, t4
Health-related quality of life	European Quality of Life Scale (EQ-5D), which comprises the EQ-5D index and the EQ-5D visual analog scale (VAS). The EQ-5D index has five domains (mobility, self-care, activities, pain/discomfort, and anxiety/depression) on a five-point scale ranging from no problems to being unable. The EQ-5D VAS measures total health status on a scale 1–100 where 100 is high	t1, t2, t3, t4
Sense of coherence	Sense of Coherence Questionnaire (SOC-13). Total score ranging from 13 to 91, where higher scores indicate better sense of coherence. Responses are measured on a seven-point Likert scale (1–7)	t1, t2, t3, t4
Positive mental health	The Mental Health Continuum Short Form (MHC-SF). The MHC-SF comprises 14 items, which are scored on a six-point scale (0–5). The summary score ranges from 0 to 70, where higher scores indicate higher levels of positive mental health	t1, t2, t3, t4
Other measures		
Age	Years	t1
Gender	Female/Male	t1
Marital status	Married/Cohabiting/Single/Widowed/Separated or divorced	t1
Living situation	Living alone/Living together with someone	t1
Level of education	Primary school/High school/1–3 years University/>4 years University	t1
Current work status	Retired/Disability benefit/Working/Sick leave/Unpaid work/Unemployed/Student	t1
Motivation for rehabilitation	Numeric scale of 1–10, where 1 is low motivation	t1
Main health challenge	The most dominant health problem	t1
Other health challenges	Presence of additional health problem(s), yes/no	t1
Health status	Changes in health status in last 10 weeks, 3.5 months, and 6 months, respectively: yes/no	t2, t3, t4
Health care services and cost measures		
Warranted community-based assistance	*Frequency and type of assistance required*	t1
	Home help/Nurse/Auxiliary nurse/Occupational therapist/Physiotherapist/Mental health service/Meals on Wheels/Other assistance/No assistance	
Inpatient and outpatient treatment since last assessment	*Frequency and type of cointerventions*	t2, t3, t4
	Hospital admissions/Admissions to other institutions/Day center placement/Outpatient treatment	
Current home-based assistance offered	*Presence and frequency of home-based assistance*	t2, t3, t4
	Home help/Nurse/Auxiliary nurse/Occupational therapist/Physiotherapist/Mental health service/Meals on Wheels/No assistance	
Current community institution-based service offered	*Type of institution-based service offered*	t2, t3, t4
	Nursing home placement long-term/Nursing home placement short-term/Day placement/Other institution placement/No institution placement	
Usage of home-based services	*Weekly time registration (in minutes) of working time during home visits*	t5
	Home help/Nurse/Auxiliary nurse/Occupational therapist/Physiotherapist/Social educator/Assistant/Student/Other	

t1 = baseline assessment, t2 = 10 weeks after baseline assessment, t3 = 6 months after baseline assessment, t4 = 12 months after baseline assessment, and t5 = daily assessment during 6 months after baseline assessment

#### Primary outcomes

The primary outcomes are activity performance and satisfaction with activity performance, which will be assessed by the COPM. The COPM measures a client’s self-perception of activity performance within nine activity areas; personal care, functional mobility, community management (self-care), paid/unpaid work, household management, play/school (productivity), and quiet recreation, active recreation, and socialization (leisure) [14]. The COPM starts with a semi-structured interview during which participants describe the activities that they consider to be important, but difficult to perform. Each activity is entered in one of the nine COPM activity categories and the importance of each activity is rated on a 10-point scale (10 = very important). The participant is then asked to choose up to five of the most important activities and to rate their performance and satisfaction with the performance of each activity on a scale from 1–10 (where a higher score reflects better performance and higher satisfaction). According to the COPM manual, a change of two points indicates a clinically relevant improvement or deterioration [14]. The Norwegian version of the COPM has been tested in terms of its psychometric properties with good results [15, 16].

#### Secondary outcomes

The study will include several secondary outcomes. Physical functioning will be measured by the Short Physical Performance Battery (SPPB). The SPPB aims to identify people at risk of functional decline, and it is a screening test for balance, walk function, and muscle strength in the lower limbs [17]. The SPPB comprises: 1) standing balance including side-by-side standing, and semi-tandem and tandem standing; 2) a walking test for four meters at regular pace; and 3) standing up and sitting down rapidly five times. For each component, the time required is recorded and converted into points (0–4), thereby giving a total score of 0–12 points. Freiberger et al. [18] conducted a systematic review and demonstrated that the SPPB has good validity, reliability, and responsiveness.

HRQoL will be measured by the European Quality of Life Scale with five dimensions (EQ-5D). EQ-5D comprises a questionnaire and a visual analog scale (VAS). The EQ-5D questionnaire has five domains (mobility, self-care, activities, pain/discomfort, and anxiety/depression) with five levels (no problems to extreme problems) [19]. According to the responses to all five dimensions, the HRQoL is calculated based on the UK tariff. The VAS scale is an indication of how individuals value their own health on a scale of 0–100.

The EQ-5D will be employed to measure quality-adjusted life-years (QALYs) for the cost-effectiveness analysis. QALYs are estimated by combining HRQoL with time and they are reported on a scale of 0–1, where 0 indicates death and 1 denotes perfect health. The EQ-5D has been translated into Norwegian, with satisfactory reliability, validity, and responsiveness among elderly people [20].

Coping will be measured using the Sense of Coherence (SOC) questionnaire. SOC-13 was developed by Antonovsky [21]. SOC-13 is self-reported and it comprises 13 items related to comprehensibility (five items), manageability (four items), and meaning (four items). The responses to all items are scored on a seven-point Likert-type scale. The total score ranges from 13 to 91, where higher scores indicate a stronger sense of coherence. SOC-13 has been tested for validity and reliability in several studies [22–26]. A systematic review concluded that the SOC scale appears to be a reliable, valid, and cross-culturally applicable instrument for measuring how people manage stress and stay well [23].

The Mental Health Continuum Short Form (MHC-SF) is designed to measure three dimensions of the positive mental health concept: emotional well-being (EWB), psychological well-being (PWB), and social well-being (SWB) [27, 28]. The MHC-SF comprises 14 items and the possible score range is 0–70. Each item is scored by rating the frequency of different feelings during the past month on a six-point scale, ranging from never (0) to every day (5). Higher scores indicate higher levels of positive mental health [27, 28]. Previous studies support the three-factor structure of the MHC-SF [29–31]. Convergent validity has been found in the subscales, which correlate well with the corresponding aspects of well-being and functioning [30], and with the scale as a whole [29]. A previous study demonstrated the high internal reliability of the whole scale as well as the EWB and PWB subscales, the adequate internal reliability of the SWB subscale, and moderate test–retest reliability [30]. The MHC-SF has been translated into Norwegian and employed successfully as a whole scale in a sample of Norwegian psoriasis patients [32].

### Sample size calculation

In an earlier study performed with older adults, the standard deviation for the primary outcome was shown to be 1.4 for COPM performance and 1.6 for COPM satisfaction [33]. The current trial is a multicenter study with 44 participating municipalities, so we expect that the variation in the COPM scores will be larger, and thus we employ a conservative estimate of 2.5 for the standard deviation. Furthermore, the allocation of participants to the intervention group or control group will not be randomized, and the number of participants in the intervention group will probably be three to four times that in the control group. We aim to detect a change of one point as statistically significant at a two-sided 5 % level and with a power of 80 %. Based on these estimates, sample size calculations indicate that we need to include 70 participants in the control group and 260 in the intervention group. Therefore, to consider the possibility of a relatively high dropout rate (up to 35 %) due to frail participants, a minimum of 107 and 400 participants will be included in the control and intervention groups, respectively.

### Statistical analysis

The data are suitable for multilevel or hierarchical modeling. Individuals are nested within municipalities and municipalities will be treated as fixed effects when mixed-effects models are applied.

### Participants

The analysis will include all participants who answer the questionnaire both at baseline and follow-up, even if they drop out of the intervention (intention-to-treat analysis). *P*-values will be two-sided and significant at *P* ≤ 0.05.

### Descriptive statistics

For each group, we will determine descriptive statistics of the sample’s sociodemographic and clinical characteristics at baseline, and the outcome measures at all time points. Mean (standard deviation) and median values (interquartile range), or numbers and percentages will be reported. Univariate analysis of covariance will be used to investigate differences between the groups at baseline and the end of intervention.

### Analysis of effectiveness

To evaluate whether the effect of the intervention varies according to the type of health problem(s), sociodemographic characteristics and home municipality will be used in linear mixed-effects models. Given the multilevel structure of the data (individuals over time within municipalities), we will control for stable differences between municipalities using a so-called fixed-effects model. Hence, we will control for time-invariant independent variables at the municipality level, such as the size of the municipality, demographics, and resources. In addition, the effect sizes will be calculated by dividing the difference of the mean change in the intervention groups and control groups by the standard deviation of the pretreatment scores.

### Analysis of cost-effectiveness

To assess the cost-effectiveness of reablement, we will need to estimate health outcomes and costs. The health outcomes will be measured using the EQ-5D and the costs will include health care sector costs. To estimate costs, we will include the cost of the intervention, which includes the hours, frequency, and type of training received in private homes according to health care professionals. Furthermore, the use of other health care services (home care services, rehabilitation, and institution) will be recorded for both the intervention and control groups over a period of 6 months. Standard methods for economic evaluation will be applied and the cost-effectiveness will be calculated as the incremental cost-effectiveness ratio, which is defined by the cost per incremental QALY. Further uncertainty will be determined by applying the bootstrap method with 1000 replicates to illustrate the variation in the patient population in terms of incremental health gains and costs.

### Ethics and dissemination

This study has been approved by the Regional Committee for Medical and Health Research Ethics for Western Norway (REK West, 2014/57). Participants will be coded and the analysis will be performed anonymously. The procedures will be conducted in accordance with the Declaration of Helsinki (1975), as revised in 2013 [34]. A declaration of voluntary participation with information about the study purposes and consequences, emphasizing the right to withdraw from the study, will be signed by each participant.

We will communicate the results in a report to the funder and in articles published in peer-reviewed journals. In addition, results will be presented to the municipalities involved in the study, health care professionals, and the public in general, through various national and international events and websites.

## Discussion

Reablement is a new and promising rehabilitation model, which is being implemented in several Western countries to meet current and future needs for home-based services [2]. However, there is limited evidence of the clinical and cost-effectiveness of this intervention.

The current trial will be a national multicenter study of reablement in Norway. To our knowledge, no previous study of reablement has included this many municipalities and participants in a nationwide clinical controlled trial. The multicenter design will ensure that the new program will be implemented in various settings, thereby enhancing the generalizability of the results.

This study has been registered with ClinicalTrials.gov (October 24, 2014) and it follows the SPIRIT 2013 statement [13], which defines standard protocol items for clinical items, thereby facilitating the transparency, proper conduct, reporting, and external review of the trial.

Patient-oriented outcome measures and procedures that help to enhance the role of the participant in the team care process as well as communication between health professionals were advocated in a review of the efficacy of multidisciplinary team care programs [35].

The primary outcome of this study will be the COPM, which is a patient-specific measure that allows each participant to choose and rate the activities that she/he considers important to perform in her/his daily life (cf. the manual [14]). The COPM captures the activities that are direct concerns for the individual, so the “noise” that is present in standardized instruments will be reduced, which theoretically has the potential to make this measure more responsive in capturing the effects of rehabilitation. In addition, the prioritized activities will be used as a basis for developing rehabilitation goals, thereby enhancing communication and giving an active role to the participants in the rehabilitation process.

Reablement is a complex intervention with a multicomponent nature; therefore, it is crucial to ensure congruence between the outcomes and the content of the intervention. Thus, several secondary outcomes are included, which have the potential to capture various effects in addition to the main outcome of COPM, such as improvement in coping and mental health. Furthermore, this study will also investigate the use of health care services and cost data, although some methodological limitations need to be discussed.

Compliance with the intervention and data collection procedures comprises a possible threat to the reliability of the study. This is a great challenge because a large number of municipalities are involved, as well as many different health care professionals. The reablement intervention is also individually tailored, which further increases the complexity. The intervention will vary between individuals, but also between municipalities, so it is important that all of the health care providers receive sufficient training and information. This will ensure that the correct way of implementing the intervention is understood and internalized, and that the data collection procedures are followed.

To ensure compliance with the study procedures, all municipalities will receive training. Furthermore, we will ensure that if a health professional leaves the study, then their replacement will receive sufficient training in both the intervention and the data collection procedures. In addition, the principal investigator will make regular contact with each municipality to ensure compliance with the procedures. The principal investigator will also check all of the incoming data material continuously to detect any misunderstandings and missing values, which will be corrected accordingly, if possible.

Another challenge is that the baseline COPM interview and scoring process might have a therapeutic effect, independent of the forthcoming interventions [36, 37]. The COPM interview may promote consciousness and motivation, and thus a process of change may also start in the control group, thereby diminishing the potential differences between the control and intervention groups.

In the present study, the allocation of participants to the intervention group or control group will not be randomized. As a consequence, its capacity to detect causal relationships will be weaker than that in a true experimental design. However, an important strength is that this study will occur in natural settings; hence, its practicality, feasibility and, to some extent, generalizability may be high. Furthermore, the controlled design will allow us to determine whether the participants in the two groups have similar baseline levels in terms of relevant clinical characteristics such as activity performance and coping [38]. If the groups are comparable at baseline, we may be relatively confident in inferring that any posttest differences are the result of reablement. In addition, if the results indicate initial differences, these may be controlled in the statistical analyses of between-group differences.

In summary, the findings of the present study may make an important contribution to our knowledge of rehabilitation approaches for community-dwelling adults. If the reablement model proves effective, it may also be applied within other fields of rehabilitation.

## Abbreviations

COPM: Canadian occupational performance measure
EQ-5D: European quality of life scale with five dimensions
EWB: Emotional well-being
HRQoL: Health-related quality of life
MHC-SF: Mental health continuum short form
PADL: Personal activities of daily living
PWB: Psychological well-being
SOC: Sense of coherence questionnaire
SPIRIT: Standard protocol items: recommendations for intervention trials
SPPB: Short physical performance battery
SWB: Social well-being
QUALY: Quality-adjusted life-year
VAS: Visual analog scale
