# Erratum to: Study protocol for a multicenter investigation of reablement in Norway

**DOI:** 10.1186/s12877-015-0115-z

**Published:** 2015-10-09

**Authors:** Eva Langeland, Hanne Tuntland, Oddvar Førland, Eline Aas, Bjarte Folkestad, Frode F. Jacobsen, Ingvild Kjeken

**Affiliations:** Centre for Care Research Western Norway, Bergen University College, P.O Box 7030, 5020 Bergen, Norway; Department of Nursing, Bergen University College, P.O Box 7030, 5020 Bergen, Norway; Departement of Occupational Therapy, Physiotherapy and Radiography, Bergen University College, P.O Box 7030, 5020 Bergen, Norway; Haraldsplass Deaconess University College, Ulriksdal 10, 5009 Bergen, Norway; Institute of Health and Society, Research Centre for Habilitation and Rehabilitation Models and Services (CHARM), Faculty of Medicine, University of Oslo, P.O Box 1089, Blindern, 0318 Oslo Norway; Department of Health Management and Health Economics, University of Oslo, P.O Box 1089, Blindern, 0318 Oslo Norway; Uni Research Rokkan Centre, P.O Box 7810, 5020 Bergen, Norway; Diakonhjemmet Hospital, National Advisory Unit on Rehabilitation in Rheumatology, P.O. Box 23, Vinderen, 0319 Oslo Norway; Oslo and Akershus University College of Applied Sciences, Program of Occupational Therapy, Prosthetics and Orthotics, P.O. Box 4, St. Olavs plass, 0130 Oslo, Norway

Unfortunately, the original version of this article [1] contained an error. The name of the author Eva Langeland was incorrectly spelt as Eva Langland. The correct spelling, Eva Langeland, has been included in the author list above.
